# The Influence of Sociodemographic Heterogeneity on the Perceptions of COVID-19: A Countrywide Survey Study in the USA

**DOI:** 10.3390/ijerph18178922

**Published:** 2021-08-25

**Authors:** Pritish Mondal, Ankita Sinharoy, Binu-John Sankoorikal, Roopa Siddaiah, Lauren Mazur, Gavin Graff

**Affiliations:** 1 Division of Pediatric Pulmonology, Department of Pediatrics, Penn State College of Medicine, Hershey, PA 17033, USA; bsankoorikal@pennstatehealth.psu.edu (B.-J.S.); rsiddaiah@pennstatehealth.psu.edu (R.S.); ggraff@pennstatehealth.psu.edu (G.G.); 2 Penn State University, Harrisburg, PA 17057, USA; axs6214@psu.edu; 3 Penn State College of Medicine, Hershey, PA 17033, USA; lmazur@pennstatehealth.psu.edu

**Keywords:** COVID-19, perception of COVID-19, preventive behavior, COVID-related stress, survey study, general linear model, sociodemographic predictors

## Abstract

**Background:** Sociodemographic factors such as age, race, education, family income, and sex have been reported to influence COVID-related perceptions, reflected by knowledge, stress, and preventive behavior. We conducted a US-based survey to estimate the difference in COVID-related perceptions among diverse sociodemographic groups and the influence of sociodemographic heterogeneity on COVID-related perceptions. **Methods:** The survey enquired about sociodemographic parameters and relevant information to measure knowledge, stress, and preventive behavior. COVID-perception scores among sociodemographic subgroups were compared with ANOVA (Bonferroni). The general linear model (GLM) was used to estimate the association among sociodemographic factors and COVID-related perceptions. **Results:** Females (75%) and White participants (78%) were the predominant (N = 3734). Females, White participants, wealthy, and educated participants demonstrated better knowledge, while participants of minority races, younger ages, low incomes, and females experienced high stress. Females, African-Americans, and educated participants better adopted preventive behaviors. Race, family income, and sex were the highest contributors to the predictive model. Sociodemographic determinants had statistically significant associations with knowledge (F-score = 7.72, *p* < 0.001; foremost predictor: race), stress (F-score = 16.46, *p* < 0.001; foremost predictor: income), and preventive behavior (GLM: F-score = 7.72, *p* < 0.001, foremost predictor: sex). **Conclusion:** Sociodemographic heterogeneity significantly influenced COVID-related perceptions, while race, family income, and sex were the strongest determinants of COVID-related perceptions.

## 1. Introduction

SARS-CoV-2, a novel coronavirus that can lead to the development of coronavirus disease (COVID-19), was declared a global pandemic by the World Health Organization on 11 March 2020 [1]. Strategies to mitigate the spread of the virus have been focused on preventive behaviors, including hand hygiene, mask-wearing, social distancing, and closing down large sectors of society at the peak of the pandemic worldwide [2]. These strategies led to unprecedented changes in social behaviors [3]. The COVID-19 pandemic has dominated the American news cycle for much of 2020 and has played a significant role in modulating behaviors [4].

The ever-changing scenario of COVID-19, along with the torrent of information, social isolation, looming uncertainty, and wide-ranging effects of COVID-19, has led to a significant degree of stress, anxiety, and depression amongst the population. Several previous studies have reported an increased psychopathological consequence among healthcare workers (HCW), especially female HCW, caring for COVID-19 patients [5,6]. Poor socio-economic conditions have also affected mental health during COVID-19 [7,8,9]. Studies from the USA as well as other countries reported that females, younger age groups, and people with unsteady income perceived higher stress during the pandemic [8,9,10]. COVID-19 outcomes in the United States reflected racial disparities; African-American participants and Hispanic participants were reported to have several-fold higher risks of hospitalization and death due to COVID-19 than White participants [11]. Thus, various communities might perceive COVID-related threats very differently.

We identified three interrelated factors that reflect COVID-related perceptions—knowledge about the disease, stress, and preventive behaviors practiced during the pandemic [12,13,14,15]. COVID-related perceptions and sociodemographic factors such as age, race, financial security, sex, and education, have complex interplay. For example, Rodríguez et al. demonstrated that young adults, women, and people of lower economic status in Spain experienced higher stress than the rest of the Spanish population during COVID-19 [16]. Likewise, a recent study suggested that the Hispanic population was less engaged in preventive behaviors, which might have resulted in a worse outcome of COVID-19 among the Hispanic community [17]. Several studies also recognized socio-economic factors influencing social behavior during the pandemic [18,19,20,21]. However, very few US-based studies compared the relative influence of sociodemographic determinants on COVID-related behaviors while considering the complex interplay among those predictors [22].

Centers for Disease Control and Prevention (CDC) had consistently emphasized adherence to preventive behaviors to prevent the spread of COVID-19 [2]. Previous studies, such as a telephonic survey of New York state residents during the H1N1 pandemic of 2009, showed that the perceived threat of the disease was a predictor of adherence to the preventive guidelines [12,23,24]. A COVID-19 survey on German participants reported higher odds of adhering to preventive behavior among people with higher education and female sex [25]. Preventive behaviors can be heterogeneous depending on the sociodemographic diversities [26,27].

To address this knowledge gap, we designed a population-based survey with a primary objective to estimate the difference in knowledge, stress, and preventive behaviors among different groups of individuals based on sociodemographic characteristics. The secondary objective was to identify the significant sociodemographic determinants of COVID-related perceptions.

## 2. Methods

1. Study design and outcome measures: We conducted an online survey between May 2020 and January 2021. The survey was built in Redcap to test three unique outcome measures related to the perception of COVID-19: knowledge, stress, and preventive behaviors [28].
  (a) COVID-related knowledge: Knowledge was assessed with several metrics, including disease spread, risk factors for severe infection, prevention, treatment, and general information. Most of the questions were on a dichotomous scale (Yes vs. No response) (Supplemental File S1).
  (b) Stress: Stress during the pandemic was assessed with seven metrics: unemployment, confinement, food availability, risk of death, risk of being infected, having access to an equipped medical facility, and the overall situation in the state of residence. Each of the stress scores was measured using a five-point Likert scale (ranging from ‘no stress’ to ‘very stressful’) (Supplemental File S1).
  (c) Preventive Behaviors: We enquired about study participants’ attitudes towards preventive recommendations, including social distancing, use of a face mask, hand washing, and lockdown using a five-point Likert scale (representing ‘strongly agree’ to ‘strongly disagree’) (Supplemental File S1).
2. Sociodemographic determinants of COVID-related perceptions: Study participants were categorized based on several sociodemographic factors: age (categorized as age-group I to V, in chronologically ascending order), race (White, African-American, Hispanic, Asian, Others), sex, level of education (assigned in ascending order: high school or below, undergraduate, graduate, masters, Ph.D./professionals (MD, MBA)), family income (four groups in ascending order), and state of residence. Fifty US states were grouped into nine divisions following the US census bureau (Table 1) [29]. HCW (Yes vs. No) and access to equipped healthcare were additionally included as independent variables.
3. Study respondents below 18 years of age and residents from countries outside of the USA were excluded from study analyses. The institutional review board approved the study protocol. The Redcap link for the public survey was sent out using several avenues: Online portals such as Researchmatch and Studyfinder [30,31], social media platforms including Facebook, and bulk e-mail generated by the ‘Marketing and Communication’ department of Hershey Medical Center. We used SAS 9.4 and SPSS 27 for statistical analyses [32,33].
4. Computation of dependent variables: To mathematically analyze response to dichotomous categorical questions (Yes vs. No) assessing knowledge and preventive behavior, we assigned a score of 1 or 0 for right or wrong answers, respectively. Likewise, most stress-related questions were framed in a Likert scale (1–5), and the scores were calculated accordingly. We computed combined scores for each of dependent variables (knowledge, stress, and preventive behavior) using ‘factor analysis’ to ease the data analyses and interpretation [34]. For example, all the metrics related to knowledge were regrouped using factor analysis into a single normalized variable (ranging between 0–10), representing a behavior score for each study participant. Similarly, parameters of stress, preventive behaviors were also redefined into 0–10 scales, respectively. We used t-tests to compare perception scores between dichotomously distributed independent variables, such as sex (male vs. female) and HCW (Yes vs. No) and ANOVA (Bonferroni posthoc test) to compare perception scores among the subcategories of other sociodemographic determinants (Supplemental Tables S1 and S2).
5. Time trend analyses: Since this survey took place over eight months and focused on a moving target (COVID-related attitudes and behavior), the associations between predictors and outcomes were potentially dynamic over the study period. Thus, to determine the time-trend of COVID-related perceptions, we used a time-series modeler in SPSS. Ljung-Box Q test [35] was conducted to test the null hypothesis that theorized “Autocorrelation does not exist between survey time period and COVID-related perceptions”.
6. Multivariate general linear model (GLM): GLM was conducted instead of multiple linear regression since we had both ordinal and categorical independent variables (sociodemographic factors) and three continuous dependent variables (COVID-related perceptions). Model effects were estimated with type III analyses, while F-statistics and *p*-values measured the strength of association between the dependent and independent variables. Pillai’s trace test [36] (value and F-statistics) was used to estimate the overall effect of the predictors on the model as higher values indicate a more substantial effect.

## 3. Results

**Figure 1:**
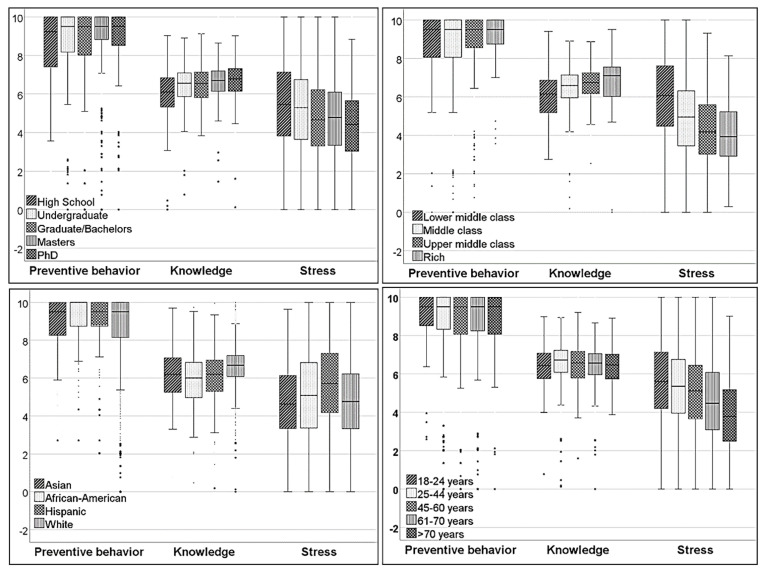
Box-plot representing differences in preventive behaviors, knowledge, and stress among different groups categorized based on education level, family income, race, and age.

1. Distribution of sociodemographic factors: We received 4183 responses, of which we included 3734 adult participants who resided in the USA. More than three-quarters of the study participants were White, and the other races represented less than 10% each (Table 1). The sex distribution was skewed, with a female to male ratio of 3:1 (Table 1). In comparison, the study participants were more evenly distributed based on age and education categories. While age-group II, III, and IV dominated the age distribution, education groups were represented mainly by undergraduate and master’s (Table 1). When the study subjects were categorized depending on their family income, more than half of the participants belonged to the middle-class, followed by the upper-middle-class group (Table 1). Among the nine US census regions, the Mid-Atlantic region (23.1%) and South-Atlantic (17.9%) were the best represented, while the New-England region had the lowest response (3.8%) (Table 1). Each of the remaining six regions had a share of 6.6% or above to the participant pool. The study cohort was comprised of 17.2% HCW.
2. Power of the study: We used the comparative analysis of the stress scores between 869 male (4.18 ± 2.04) and 2625 female participants (5.17 ± 2.02) to estimate the power of the study. With an alpha = 0.05, the projected power of the study was 1.
3. Time trend analyses: Ljung-Box Q test did not favor correlations between time-factor and COVID-related perceptions. The *p*-values (Ljung-Box Q test) for knowledge, stress, and preventive behavior were not statistically significant (0.185, 0.127, and 0.372, respectively), which excluded significant drifts in COVID-related perceptions over the study period.
4. COVID-perception scores and subcategories of sociodemographic factors:
  (a) COVID-related knowledge: Female participants (6.59 ± 1.20) demonstrated better knowledge than the males (6.35 ± 1.27) (*t*-test: *p* < 0.001) and HCW had achieved higher knowledge scores (6.79 ± 1.25) compared to non-HCW (6.47 ± 1.20) (*t*-test: *p* < 0.001). White participants demonstrated better knowledge questions than minority race groups (Table 2, Figure 1). There was no difference among various age groups (Supplemental Table S1). Knowledge scores also steadily increased with higher education and family income (Table 3, Figure 1) (Supplemental Table S2).
  (b) COVID-related stress: Female participants (5.17 ± 2.02) experienced higher stress levels (*t*-test: *p* < 0.001) compared to male participants (4.18 ± 2.04), while HCW (5.38 ± 1.93) reported higher stress compared to non-HCW (4.84 ± 2.10) (*t*-test, *p* < 0.001). White participants reported lesser stress compared to African-American and Hispanic participants (*p*-value of 0.08, <0.001, respectively). Hispanic participants also demonstrated higher stress compared to African-American and Asian participants (*p* = 0.07 and *p* < 0.001, respectively) (Figure 1, Supplemental Table S1). The level of stress steadily declined with an advance in age, higher education, and income (Figure 1, Table 2 and Table 3, Supplemental Tables S1 and S2). Family income and education levels also had a significant association (Pearson Chi-Square = 421.87, *p* < 0.001).
  (c) Preventive behavior: Female participants (8.94 ± 1.63) showed better adoption of preventive guidelines compared to the males (8.18 ± 2.20) (*t*-test: *p* < 0.001). However, there was no significant difference between HCW (8.80 ± 1.75) and non-HCW (8.73 ± 1.84) (*t*-test: *p* = 0.373). Among various race groups, African-American participants had the best attitude towards preventive guidelines, and their scores were significantly higher than White participants (Table 2, Figure 1), but not compared to other races, based on ANOVA (Bonferroni). The was no difference among the various age groups (Supplemental Table S1). Survey participants with higher income and education consistently demonstrated a better acceptance of preventive guidelines (Table 3) (Supplemental Table S2).
5. GLM for COVID-related perceptions:

Based on Pillai’s trace test (value), the race was the predominant contributor to the GLM, followed by family income and sex, respectively (Table 4). Please review Supplemental Table S3 for parameter estimates in GLM.

(a) Knowledge: The prediction model was statistically significant (*F*-statistics = 12.42, *p* < 0.001). In descending orders, race, family income, and education level were the top predictors, followed by age and sex (Table 4).

(b) Stress: The model was statistically significant (*F*-statistics = 16.64, *p* < 0.001). Family income was the strongest predictor of stress, followed by sex and age. Among the other predictors, hospital access, healthcare workers, and education level also contributed significantly (Table 4).

(c) Preventive behaviors: The GLM had superior performance compared to the null model (*F*-statistics = 7.72, *p* < 0.001). Sex was the strongest predictor of preventive behavior, followed by education level and race. Age, US region, and healthcare access also contributed significantly to the model (Table 4).

## 4. Discussion

Our study estimated the relative importance of sociodemographic factors which influenced stress, knowledge, and preventive behavior among the US participants during the pandemic. Thus, the report will help in better strategic planning to address the multifaceted problem with COVID-related perception. The study analyses identified race, family income, and sex as the key determinants. We observed a higher response rate from females than males, which perhaps reflected a general trend of females’ higher response to online surveys [37]. Several previous studies have reported a higher stress level and other psychosocial effects in females than males during this pandemic [8,38,39]. Likewise, sex was the strongest predictor of stress in our study population, and that possibly increased the likelihood of females accepting preventive guidelines and seeking COVID-related knowledge.

As per recent literature, COVID-19 perceptions are influenced by social inequalities [40]. People with lower income, lack of higher education, and African-American participants had lower knowledge of COVID-19 [41]. Moreover, minority races (African-American and Hispanic participants) and lower-income populations were reported to be at higher risk of mortality [42]. While African-American and Hispanic participants had a four times higher risk of getting hospitalized and 2.8 times higher risk of mortality [43], they were also more likely to lose their job and insurance through work, which decreased their access to healthcare facilities [44]. Thus, some members of the minority communities were under significant strain while battling financial crises, loss of insurance, and a higher risk of morbidity and mortality due to COVID-19. Certainly, this marginalized population within society is at increased risk for psychosocial stress [45]. Despite the perceived threats, African-American participants had a lower knowledge score than White participants, likely due to their lower educational status (undergraduate vs. graduate for African-American vs. White participants, *p* < 0.001 Mann–Whitney). Jones et al. reported a similar disparity in COVID-related knowledge among various race groups [22]. GLM determined ‘race’ as the most important contributor to the multivariate prediction model of COVID-related perceptions, reflecting the impact of racial disparity during the pandemic (Table 4).

People aged 55 years and above have died disproportionately (92.7%) of COVID-related complications. However, recent studies have reported that adults under 25 years experienced the highest stress during the pandemic [46,47]. We found a similar trend, which underscored that the risk of death was not the only stressor. Several additional factors such as lack of social interaction, job loss, financial difficulties, and concern with vulnerable family members had substantially impacted the mental health, especially in the younger age groups [48,49]. The youngest survey participants also had the highest acceptance of the preventive guidelines, perhaps influenced by their stress (Table 2). The health care profession was assumed to be a determinant of a perceived threat of contracting or dying from the disease. Interestingly, HCW, compared to non-HCW, did not have a difference in preventive behavior, despite higher stress scores and better knowledge.

Family income and education levels had a significant mutual association. Both the factors demonstrated an inverse correlation with COVID-related knowledge and stress, as the participants with higher education and income possibly were less unsecured with finance, job, and other basic necessities. Higher education and income were the strongest predictors of COVID-related knowledge. This, perhaps, positively influenced their attitude towards adopting preventive behaviors.

Our study has several limitations. Since the study participants were recruited randomly using available means, the study cohort should be considered as a non-representative sample. However, Researchmatch, the primary recruitment method, offered 150,000 volunteers to reach out randomly [30]. Thus, the study participants were relatively evenly distributed throughout the country. While knowledge and preventive behaviors had reasonable objective measurements, the stress level assessment might have been influenced by individuality. Since this was an online survey, we could only reach the population who are internet-savvy, and a selection bias could not be excluded. However, that is the nature of any online survey. Recent market surveys showed that females use social media more than males, and people with low income are less likely to use social media than the affluent class [50]. We recruited a large number of participants via social media. Thus, the female and middle-class dominant study participants perhaps reflected that trend. Since this was an uncompensated survey, participants like HCW, or someone with a COVID-19 case in the family, could be keener to respond than the rest of the population.

Nonetheless, our study has a few strengths. The survey respondents constitute a diverse population with a representative of all US states. A similar study illustrating the relative importance and interaction among sociodemographic determinants of COVID-related behavior and perceptions has not been reported to the best of our knowledge. Recently CDC has underscored that the impact of health inequity on people of minority races needs to be addressed [51]. Additionally, the preexisting inequality in access to higher education, family income, and social security widened between racial minority communities and White participants during the pandemic [52]. The study results highlighted the factors contributing to the disproportionate impact of the pandemic on people of lower socio-economic status. Thus, the results may help inform future strategies to mitigate higher psychosocial stress among the racial minority communities and targeted intervention protecting the vulnerable population. Our study emphasized that, in spite of the lack of knowledge of COVID-19, African-American and Hispanic participants had better adherence to the preventive behavior, which could be related to increased stress. Certainly, current national health education policies were more useful among the White populations [22], and social awareness strategies focusing on education and engagement of racial minorities perhaps would have led to a better outcome of COVID-19. For example, recent studies show that, despite increased COVID-19 vaccination across the country, African-American and Hispanic participants are still lagging in vaccine acceptance due to increased hesitancy [53]. Equity in COVID-19 response is critical for the nation’s overall health and economic prosperity, and the federal government has undertaken a wide range of initiatives to advance health literacy among racial minorities [54]. However, social and economic inequity is a longstanding challenge, and to overcome efforts from all stakeholders, including states, local, public and private organizations, would be necessary. This study elucidates expected behavior from various sections of the US societies during the pandemic, influenced by their socio-economic status. Finally, the large sample size and reproducibility of earlier results should also be considered strengths of this study.

## 5. Conclusions

Sociodemographic factors were significantly associated with COVID-related perceptions. Race was the most significant determinant, followed by family income and sex. While female participants compared to males had higher knowledge and stress, and demonstrated better attitudes towards preventive guidelines, the younger participants were vulnerable to the psychosocial impacts of the pandemic. In addition, participants with lower education and income experienced higher stress and had lesser knowledge than the remainder of the study population.

## Figures and Tables

**Table 1:** Characterization of the Sociodemographic predictors of COVID-related perceptions.

Sociodemographic Predictors		N	Percent	Sociodemographic Predictors		N	Percent
**Sex**	Female	2625	75.1%	**Race**	Asian	157	4.6%
	Male	869	24.9%		African-American	228	6.7%
**Family Income**	Lower-middle class	496	14.1%		Hispanic	219	6.4%
	Middle class	2067	58.6%		White	2673	78.0%
	Upper-middle class	892	25.3%		Others	150	4.4%
	Rich	73	2.1%	**Age**	Group I (18–24 years)	187	5.3%
**US Region**	East North Central	507	14.6%		Group II (25–44 years)	1079	30.6%
	East South Central	266	7.7%		Group III (45–60 years)	888	25.2%
	Mid-Atlantic	799	23.1%		Group IV (61–70 years)	837	23.8%
	Mountain	253	7.3%		Group V (>70 years)	533	15.1%
	New England	130	3.8%	**Education Level**	High School	407	11.6%
	Pacific	423	12.2%		Undergraduate	953	27.2%
	South Atlantic	620	17.9%		Graduate	768	21.9%
	West North Central	237	6.8%		Masters	850	24.3%
	West South Central	229	6.6%		Ph.D./Professionals	524	15.0%

**Table 2:** The difference in preventive behaviors, knowledge, and stress among various races and age-groups. The level of significance was estimated by ANOVA.

COVID-Related Perceptions	Race	Mean ± SD	*F*-Value (Significance)	Age	Mean ± SD	*F*-Value (Significance)
**Knowledge**	White	6.67 ± 1.13	36.05 (<0.001)	18–24 years	6.45 ± 1.31	3.00 (0.017)
	African- American	5.87 ± 1.44		25–44 years	6.63 ± 1.27	
	Hispanic	6.01 ± 1.46		45–60 years	6.51 ± 1.27	
	Asian	6.16 ± 1.44		61–70 years	6.51 ± 1.11	
	Others	6.24 ± 1.29		>70 years	6.40 ± 1.12	
**Stress**	White	4.82 ± 2.05	11.80 (<0.001)	18–24 years	5.54 ± 1.98	52.81 (<0.001)
	African- American	5.20 ± 2.29		25–44 years	5.38 ± 2.03	
	Hispanic	5.73 ± 2.16		45–60 years	5.10 ± 2.05	
	Asian	4.86 ± 2.01		61–70 years	4.64 ± 2.08	
	Others	5.24 ± 2.09		>70 years	3.94 ± 1.94	
**Preventive Behaviors**	White	8.72 ± 1.85	5.67 (<0.001)	18–24 years	8.93 ± 1.57	1.27 (0.28)
	African- American	9.22 ± 1.20		25–44 years	8.75 ± 1.82	
	Hispanic	9.07 ± 1.48		45–60 years	8.70 ± 1.86	
	Asian	8.85 ± 1.54		61–70 years	8.75 ± 1.86	
	Others	8.71 ± 1.82		>70 years	8.71 ± 1.84	

**Table 3:** The difference in preventive behaviors, knowledge, and stress among various groups categorized based on family income and education level. The level of significance was estimated by ANOVA.

COVID-Related Perceptions	Family Income Groups	Mean ± SD	*F*-Value (Significance)	Education Level Groups	Mean ± SD	*F*-Value (Significance)
**Knowledge**	Lower middle class	5.97 ± 1.39	40.46 (<0.001)	High School	6.04 ± 1.41	24.67 (<0.001)
	Middle class	6.57 ± 1.18		Undergraduate	6.50 ± 1.21	
	Upper middle class	6.73 ± 1.08		Graduate	6.47 ± 1.16	
	Rich	6.81 ± 1.62		Masters	6.71 ± 1.13	
				Ph.D./Professionals	6.78 ± 1.18	
**Stress**	Lower middle class	5.98 ± 2.18	69.84 (<0.001)	High School	5.43 ± 2.33	18.02 (<0.001)
	Middle class	4.94 ± 2.04		Undergraduate	5.19 ± 2.14	
	Upper middle class	4.37 ± 1.92		Graduate	4.80 ± 2.09	
	Rich	4.01 ± 1.96		Masters	4.79 ± 1.91	
				Ph.D./Professionals	4.45 ± 1.92	
**Preventive Behaviors**	Lower middle class	8.67 ± 1.93	2.82 (0.038)	High School	8.30 ± 2.18	14.62 (<0.001)
	Middle class	8.71 ± 1.83		Undergraduate	8.73 ± 1.84	
	Upper middle class	8.88 ± 1.74		Graduate	8.61 ± 1.86	
	Rich	9.03 ± 1.50		Masters	9.05 ± 1.53	
	Lower middle class	5.97 ± 1.39		Ph.D./Professionals	8.91 ± 1.65	

**Table 4:** The general linear model estimated the association between sociodemographic determinants and COVID-related perceptions (knowledge, stress, and preventive behavior). Pillai’s Trace test determined the level of significance of each contributor to the model. (*p* < 0.05 is considered statistically significant).

	COVID-Related Perceptions	Type III Sum of Squares	*F*-Statistics	*p*-Value	Pillai’s Trace Test		
					Value	*F*-Statistics	*p*-Value
**Adjusted Model**	Knowledge	546.5	12.42	<0.001	N/A	N/A	N/A
	Stress	2000.42	16.46	<0.001			
	Preventive behaviors	718.8	7.72	<0.001			
**Race**	Knowledge	179.61	26.95	<0.001	0.068	13.43	<0.001
	Stress	35.85	1.95	0.083			
	Preventive behaviors	156.14	11.06	<0.001			
**Family Income**	Knowledge	70.71	17.68	<0.001	0.051	16.92	<0.001
	Stress	364.12	32.96	<0.001			
	Preventive behaviors	14.28	1.69	0.168			
**Sex**	Knowledge	19.1	7.16	0.001	0.049	24.44	<0.001
	Stress	234.69	31.87	<0.001			
	Preventive behaviors	266.79	47.26	<0.001			
**Age**	Knowledge	45.2	8.48	<0.001	0.043	10.48	<0.001
	Stress	226.88	15.41	<0.001			
	Preventive behaviors	27.76	2.46	0.044			
**Education Level**	Knowledge	70.22	13.17	<0.001	0.039	9.63	<0.001
	Stress	38.84	2.64	0.032			
	Preventive behaviors	129.27	11.45	<0.001			
**Hospital Access**	Knowledge	5.4	1.01	0.4	0.031	7.53	<0.001
	Stress	232.86	15.81	<0.001			
	Preventive behaviors	31	2.75	0.027			
**US Region**	Knowledge	60.02	5	<0.001	0.028	3.03	<0.001
	Stress	52.72	1.59	0.112			
	Preventive behaviors	58.21	2.29	0.015			
**Healthcare Worker**	Knowledge	2.42	0.91	0.403	0.007	3.60	0.001
	Stress	59.54	8.08	<0.001			
	Preventive behaviors	3	0.53	0.588			

## Data Availability

Raw data with detailed description is available online: https://data.mendeley.com/datasets/892g33m7hh/1 (accessed on 1 July 2021).

## Supplementary Materials

The following are available online at https://www.mdpi.com/article/10.3390/ijerph18178922/s1, Table S1: Comparative analyses of knowledge, stress, and preventive behavior among different races and age groups with ANOVA. Table S2: Comparative analyses of knowledge, stress, and preventive behavior with ANOVA, among various groups categorized by education level and family income. Table S3: Parameter estimates for the sociodemographic determinants. File S1: Survey questions.
