# Supplementary material for: The Influence of Sociodemographic Heterogeneity on the Perceptions of COVID-19: A Countrywide Survey Study in the USA
Source: Int J Environ Res Public Health. 2021 Aug 25;18(17):8922. doi: 10.3390/ijerph18178922 (PMC8431068; doi:10.3390/ijerph18178922)
Supplement: Supplementary file 1 [file ijerph-18-08922-s001.zip › Supplemental File 1_Survey questions.pdf]

Preventive behavior

1) Do you follow the recommendations to maintain ‘social distancing’?
   i) Strongly agree
   ii) Agree
   iii) Neutral
   iv) Disagree
   v) Strongly disagree

2) Do you follow the recommendations to maintain ‘Lock down’?
   i) Strongly agree
   ii) Agree
   iii) Neutral
   iv) Disagree
   v) Strongly disagree

3) Do you follow the recommendations for ‘Hand washing for 20 seconds’ whenever applicable?
   i) Strongly agree
   ii) Agree
   iii) Neutral
   iv) Disagree
   v) Strongly disagree

4) Do you use ‘Face mask’ in public?
   i) Strongly agree
   ii) Agree
   iii) Neutral
   iv) Disagree
   v) Strongly disagree

5) One should go to Emergency/doctor immediately if he/she has symptoms like COVID-19?
   i) Strongly agree
   ii) Agree
   iii) Neutral
   iv) Disagree
   v) Strongly disagree

Knowledge

Who are at high risk for serious coronavirus infection?

1. Elderly people (age >60 years)
i) Yes
ii) No

2. Children (0-5 years)
i) Yes
ii) No

3. Smokers
i) Yes
ii) No

4. Diabetic patients

i) Yes
ii) No

5. People without ability to fight infection
i) Yes
ii) No

6) Pregnant women

i) Yes
ii) No

7) Most COVID-19 patients need hospitalization

i) Yes
ii) No

8) One in five COVID-19 patients die

i) Yes
ii) No

9) It takes 2-3 weeks to have disease symptoms after getting coronavirus

i) Yes
ii) No

10) Before COVID-19, we did not know that coronavirus can affect human

i) Yes
ii) No

11) We can get COVID-19 from animals

i) Yes
ii) No

12) Every person in the community should be tested for COVID-19 radio (Matrix)

i) Yes
ii) No

Which of the following helps to prevent COVID-19?

13) Hand washing

i) Yes
ii) No

14) Hot and humid climate

i) Yes
ii) No

15) Exposure to bright sunlight

i) Yes
ii) No

16) Saline nasal spray

i) Yes
ii) No

17) Drinking hot beverages or alcohol

i) Yes
ii) No

18) Hand sanitizer containing alcohol radio

i) Yes
ii) No

19) Baby wipes

i) Yes
ii) No

20) COVID-19 symptoms include (CHECK ALL THAT APPLY) :

i) Fever
ii) Cough
iii) Shortness of breath
iv) Body ache
v) Sore throat

21) Vaccine

i) Pneumonia vaccine offers some protection against COVID-19
ii) Flu shot offers some protection against COVID-19
iii) Both 1 and 2 are true
iv) Both 1 and 2 are false

22) Protective measures (CHECK ALL THAT APPLY)

i) Face-mask prevents the spread of COVID-19
ii) Rubber gloves should be used while going outside (such as groceries)

23) Social distancing means keeping ____feet gap between two persons:

i) 3 feet
ii) 6 feet
iii) 10 feet
iv) 15 feet

24) If you had contact with a COVID-19 patient, you should be isolated for:

i) One week
ii) Two weeks
iii) Three weeks
iv) Four weeks

25) Coronavirus can live on a surface for:

i) Up to 6 hours
ii) Few hours to 3 days
iii) Up to 14 days

26) COVID-19 usually spreads via (CHECK ALL THAT APPLY):

i) Contact (by touch)
ii) Droplet (such as sneeze)
iii) Water (drinking water, shower)
iv) Wind/air

27) Hydroxychloroquine (Plaquenil) is useful to treat COVID-19

i) Strongly agree
ii) Agree
iii) Neutral
iv) Disagree
v) Strongly disagree

Stress

Stress Related to COVID-19 Pandemic

1) Being confined at home with minimal social interaction is

i) Very stressful
ii) Stressful
iii) Sometimes stressful
iv) Mostly handled well
v) Not stressful at all

2) Job situation or possibility of financial instability is

vi) Very stressful
vii) Stressful
viii) Sometimes stressful
ix) Mostly handled well
x) Not stressful at all

3) The possibility of getting infected with COVID-19 is

i) Very stressful
ii) Stressful
iii) Sometimes stressful
iv) Mostly handled well
v) Not stressful at all

4) Possible shortage of food and other essentials in the coming months

i) Very stressful
ii) Stressful
iii) Sometimes stressful
iv) Mostly handled well
v) Not stressful at all

5) One of your family members is at risk of serious complication due to COVID-19

i) Very stressful
ii) Stressful
iii) Sometimes stressful
iv) Mostly handled well
v) Not stressful at all

6) Lack of access to a good medical facility

i) Very stressful
ii) Stressful
iii) Sometimes stressful
iv) Mostly handled well
v) Not stressful at all

7) Situation of COVID-19 at your state

i) Very stressful
ii) Stressful
iii) Sometimes stressful
iv) Mostly handled well
v) Not stressful at all
